# Fogging With Peracetic Acid in Schools and Kindergartens

**DOI:** 10.3389/fpubh.2021.697917

**Published:** 2021-09-17

**Authors:** Ewelina Kruszewska, Henryk Grześ, Piotr Czupryna, Sławomir Pancewicz, Monika Groth, Mulugeta Wondim, Anna Moniuszko-Malinowska

**Affiliations:** ^1^Department of Infectious Diseases and Neuroinfections, Medical University of Bialystok, Bialystok, Poland; ^2^Microbiology Laboratory, University Clinical Hospital in Bialystok, Bialystok, Poland

**Keywords:** peracetic acid, fogging, decontamination, fumigation, automated

## Abstract

Disinfection is a key element in controlling infections. Fogging, also known as fumigation, is one of the most effective chemical disinfection methods. Peracetic acid (PAA) is a powerful oxidant with bactericidal and fungicidal properties. The aim of this study is to determine the type of bacteria and fungi present in educational institutions and whether disinfection by PAA fumigation in these institutions is also effective and useful, as demonstrated previously in healthcare centers. This study was carried out in five kindergartens and five primary schools in Bialystok, Poland. Three rooms have been selected in each of these educational institutions, and the disinfection was carried out in 30 rooms in total. Fogging with PAA was performed in selected rooms. Before and after disinfection, samples were collected from four surfaces: walls, tables, doors, and chair backs. Most frequently detected microorganisms in schools and kindergartens were *Micrococcus luteus (M. luteus), Staphylococcus warneri (S. warneri), Paracoccus yeei (P. yeei), Staphylococcus hominis* ssp. *hominis (S. hominis), Kocuria rhizophila (K. rhizophila), Kocuria rosea (K. rosea)*. In addition, *Staphylococcus haemolyticus (S. haemolyticus), Acinetobacter lwoffii (A. lwoffii), Kocuria kristinae (K. kristinae), Lactococcus lactis* ssp. *lactis (L. lactis)* were the most prevalent in kindergartens, whereas *Kytococcus sedentarius (K. sedentarius)* was the most prevalent in schools. Comparison of the bacterial flora of schools and kindergartens showed statistically significant differences in the prevalence of bacteria on different surfaces. A significant decrease in the number of colonies after disinfection was observed on all surfaces (*p* < 0.05). In addition, the calculated effectiveness of disinfection was 99.7% in kindergartens and 99.3% in schools. The results indicate that fogging of PAA is a highly effective method of surface disinfection in kindergartens and schools.

## Introduction

There are three ways to combat pathogens in disinfection: physical, chemical, and thermal-chemical methods. One of the most common and efficient chemical disinfection methods is the fogging process.

In fogging, an appropriate aerosol generator is used to produce the fog. The disinfectant evaporates under the influence of high temperature (60°C) and then condenses upon contact with the cooler air in the room, forming a droplet of a diameter of approximately 0.5 μm. The resulting fog may last longer than the cold fog, and this allows the chemical agent to remain in contact with the target microorganism for a longer period. Therefore, less disinfection chemical is needed, making it safer for people and the environment. Currently, alternative disinfection technologies, such as fumigation, are used in the healthcare sector to complement manual disinfection and have proven effective in reducing the environmental pollution (1, 2).

A good disinfectant should possess two basic properties: resistance to unfavorable environmental conditions (durability, good solubility, no discoloration, and corrosion on the surfaces used) and effectiveness against microorganisms (high activity against all microorganisms, no resistance even with prolonged use). Because of the environmental protection reasons, the disinfectant must not be toxic to living organisms, and it should be biodegradable quickly and easily (3). Peroxygen compounds are often used in disinfectants. These include peracetic acid (PAA), potassium persulfate, or hydrogen peroxide (H_2_O_2_) (2). Microorganisms do not develop resistance to these compounds, which is a very important feature (4).

The method of obtaining PAA was described for the first time by Freer P and Novy FG in the American Chemical Journal in 1902. Moreover, they also described a fast and broad spectrum of biocidal activity (bacteria, fungi, viruses, bacterial spores, and mycobacteria) of PAA (5). The mechanism of action of PAA is based on the oxidation of -SH groups of proteins to disulfide bridges and oxidation of double bonds occurring in the cell membrane. The destruction mechanism of the microorganism is based on the release of active oxygen, which destroys proteins, fats, and nucleic acids (4). An additional advantage of using PAA is the fact that it is harmless to the environment and is active at low concentrations, which is very useful in conditions requiring penetration of the disinfectant into porous surfaces or crevices.

According to the US Centers for Disease Control and Prevention (CDCP), PAA can cause corrosion of galvanized iron, steel, bronze, brass, and copper, but this effect can be reduced, among others, by changes in pH (6). Chemical agents based on PAA have been used in combating endemic outbreaks in medical facilities, in endoscopes, underwear, and medical tools disinfection (7). Dozens of formulations containing PAA have been registered with the United States Environmental Protection Agency as disinfectants for COVID-19 (8).

Usually, decontamination is performed with chemical disinfectants containing quaternary ammonium, peroxygen, chlorine, alcohols, or alkylamines compounds. Electronic equipment and complicated medical equipment cannot be cleaned with traditional cleaning methods. Hence, fumigation methods such as fogging are increasingly used.

The aim of this study is to assess the effectiveness of the chemicals in the fumigation process in populated classrooms. In addition, the identification of the most frequent bacteria and fungi species in the kindergarten and environment of the school was performed.

## Materials and Methods

The study was approved by Bioethical Committee of the Medical University of Bialystok, Poland.

### Choosing a Place to Conduct a Study

The study was conducted in five kindergartens and five primary schools in Bialystok, Poland. Three rooms, in which the children previously stayed during the classes, were selected in each of these educational institutions. In total, disinfection was carried out in 30 rooms. Samples were taken from each room before and after disinfection. Kindergartens are attended by children aged from 3 to 6 years, whereas primary schools are attended by children and adolescents aged from 7 to 15 years.

### Before Disinfection

Prior to the fogging process, the ventilation has been disabled, all the vents have been sealed and the doors have been taped to prevent the disinfectant from exuding the room during fumigation.

Samples for microbiological testing were collected from four different surfaces inside each room. These surfaces included tables, back of the chairs, doors, and walls. The first three surfaces were selected as the most frequently touched surfaces in the room, and the walls were chosen to test the effectiveness of long-distance disinfection.

Samples for microbiological testing were taken with an applicator (BIOMAXIMA, Lublin, Poland) and RODAC contact plates (OXOID, Deutschland Gmbh, Wesel, Germany) with a 25 cm^2^ contact surface area. The substrate on the plates allows the growth of bacteria and fungi as it contains substances that inactivate surface disinfectants. For the standardized collection of cleanliness, samples from flat surfaces using the impression method, an applicator is used. Standardization of the pressure of 500 g and the sampling time of 10 seconds minimize the occurrence of human error during the test. Thus, the results obtained with an applicator are reliable regardless of the person conducting the test. A total of 120 impressions were collected prior to disinfection.

### Fogging (Fumigation)

For research purposes, the fully automatic Aerosept 500 apparatus (Laboratories ANIOS, Lille-Hellemmes, France) was selected. It is a mobile decontamination system that uses a disinfectant in the form of a dry fog. The device was placed in the corner of the room which was being disinfected. The diffusion time depends on the volume, so the size of the room has been programmed on the device. The cubature was calculated according to the formula: height × length × width. The Aseptanios disinfectant AD (Laboratoires ANIOS, Lille-Hellemmes, France), containing PAA stabilized with an acetic acid (AA) and H_2_O_2_, was used for the fogging process. Fogging was carried out in the afternoon, immediately after the pupils left the premises. Depending on the size of the room, cold decontamination takes up to 3.5 h.

### After Disinfection

Immediately after the fumigation, all windows and doors have been opened to allow the evaporation of the disinfectant. After 30 min of airing the room, samples for the microbiological test were collected once again from the same surfaces, i.e., tables, back of the chairs, doors, and walls. After disinfection, a total of 120 samples were taken.

After the collection, the plates were incubated for 48 h at 35 ± 2°C. Afterward, the number of bacterial colonies was counted directly from the RODAC plates, and their number was given in Colony Forming Units per 25 cm^2^ (CFU/25 cm^2^).

### Identification of Microorganisms

Microorganisms of different morphology were isolated on a set of media containing the following:

- Columbia Agar with 5% Sheep Blood (OXOID, Basignstoke, United Kingdom).
- Mac Conkey medium with the addition of crystal violets that inhibits the growth of gram-positive cocci and lactose (OXOID, Basignstoke, United Kingdom).
- Mannitol Salt Agar; Chappman (OXOID, Basignstoke, United Kingdom)
- Sabouraud medium with the addition of chloramphenicol and gentamicin (GRASO, Jabłowo, Poland).

The inoculated media were incubated in aerobic condition at 35°C ± 2°C.

Based on the morphology of the colonies, the cultured microorganisms were first differentiated by: (I) gram staining; (II); detection of catalase production; and (III) detection of cytochrome oxidase production, then the identification of species or genus was made.

#### Gram Staining

The bacteria collected from the selected colony were suspended on the glass slide coated with 100 μl of 0.45% NaCl solution. The slide was then allowed to dry at the room temperature. The dried slide was then fixed over the flame of an alcohol burner and stained with a Gram stain kit from Becton Dickinson (Sparks, Maryland, USA).

1. Crystal violet 3 min.
2. Lugol's solution 1.5 min.
3. Alcohol decolorizing agent 30 sec.
4. Safranin 20 sec.

The stained slide was assessed under a ×100 objective and ×20 eyepiece immersion microscope Olympus CH20 (Olympus Optical Co, Ltd., Japan). Based on the obtained image, the staining (gram-positive or gram-negative), shape (cocci, bacilli), and arrangement (clusters, chains, pairs, etc.) were determined.

#### Determination of Catalase Production

Catalase was detected with 3% H_2_O_2_. The bacteria taken from the culture on the blood medium were placed on the glass slide, spread in an elliptical shape, and sprinkled with H_2_O_2_. The appearance of foaming was assessed.

#### Cytochrome Oxidase Assay

Oxidase was determined with Oxidase strips (OXOID, Adelaide, Australia) by applying the bacterial colony with a plastic loop to the strip and observing the appearance of the color, with the purple color indicating the positive reaction.

After the initial division of the bacteria into gram-positive and gram-negative, they were identified with the VITEK 2.0 automatic system (bioMérieux, Craponne, France). Tests were performed in accordance with the instructions of the system manufacturer, using appropriate cards.

According to the instructions of the manufacturer, the bacterial suspension in 0.45% NaCl solution with a turbidity of 0.5 degrees McFarland (0.50–0.63°MF) was made.

Mold fungi were identified in the direct preparation based on morphological features, and yeast-like fungi were identified from the suspension with a turbidity of 2°MF (1.80–2.20°MF) using VITEK 2 YST cassettes (bioMérieux, Craponne, France).

### Statistical Analysis

The data were statistically analyzed using the Statistica 13.0 software (StatSoft Inc., Krakow, Poland) *p* < 0.05 were considered statistically significant. Mann Whitney *U* test and Wilcoxon signed-rank test were used for the analysis.

## Results

Most frequently detected microorganisms in schools and kindergartens included *Micrococcus luteus (M. luteus), Staphylococcus warneri (S. warneri), Paracoccus yeei (P. yeei), Staphylococcus hominis* ssp. *hominis (S. hominis), Kocuria rhizophila (K. rhizophila), Kocuria rosea (K. rosea)*. In addition, *Staphylococcus haemolyticus (S. haemolyticus), Acinetobacter lwoffii (A. lwoffii), Kocuria kristinae (K. kristinae), Lactococcus lactis* ssp. *lactis (L. lactis)* were the most prevalent in kindergartens, whereas *Kytococcus sedentarius (K. sedentarius)* was the most prevalent in schools.

Comparison of the bacterial flora of kindergartens and schools using the Mann Whitney *U* test and Wilcoxon signed-rank test showed a statistically significant difference. In kindergartens, the more frequent presence of the following bacteria has been shown on:

- walls: *S. warneri* (*p* < 0.001) and *S. haemolyticus* (*p* = 0.007)
- tables: *K. rosea* (*p* = 0.03) and *S. haemolyticus* (*p* = 0.02)
- chair backs: *S. warneri* (*p* = 0.02) and *A. lwoffii* (*p* = 0.04)
- door: no differences found
  and *P. yeei* was more common in schools (*p* = 0.04).

The disinfection effectiveness was calculated separately for walls, tables, chair backs, and doors by comparing the mean number of bacterial colonies (CFU/25 cm^2^) before and after disinfection in both the institutions. The data and the results are presented in Table 1.

**Table 1:** Disinfection effectiveness on various surfaces.

	**Kindergartens**				**Schools**			
	**Mean number of colonies (CFU/25cm^2^)**			**Effectiveness (%)**	**Mean number of colonies (CFU/25 cm^2^)**			**Effectiveness (%)**
	**Before disinfection**	**After disinfection**	***P***		**Before disinfection**	**After disinfection**	***p***	
Overall	135.92	0.47	<0.05	99.7	82.19	0.57	<0.05	99.3
Chair backrest	146.73	0.14	<0.05	99.2	79.67	0.67	<0.05	99.2
Tables	256.86	0.50	<0.05	99.8	141.20	0.67	<0.05	99.5
Walls	70.53	0.29	<0.05	99.6	42.27	0.60	<0.05	98.6
Doors	67.53	1.00	<0.05	98.5	64.43	0.33	<0.05	99.5

## Discussion

To the best of our knowledge, this is the first study assessing the effectiveness of fogging with PAA in schools and kindergartens. Peracetic acid is an excellent bactericide and fungicide, used mostly in the environment of the hospital. Fogging was proven to be a good method in the removal of contamination in hospitals and healthcare-related places. Ali et al. established that the use of air-borne H_2_O_2_ gives excellent results in surgical wards, single isolation rooms, and bathrooms (9). Another study proved that decontamination by dry fogging with H_2_O_2_ in dental surgery has a high potential as H_2_O_2_ fogging resulted in a significant decrease in the number of bacterial and fungal colonies present in these settings (10). Moreover, fogging demonstrated good compatibility with electronic sensors and facilities used in laboratories, therefore, there is no risk of damaging the equipment (11, 12). Recent studies showed that fogging is also very effective in the removal of SARS-CoV-2 from various surfaces. In their study, Cutts et al. demonstrate that dry fogging with PAA completely inactivated SARS-CoV-2 on test surfaces. They also suggested that with the ease of use, low cost, and overall effectiveness of a PAA, dry fogging should also be considered for decontamination in the prevention of COVID-19 (13). Another study demonstrated the effectiveness of PAA for disinfection of *Geobacillus stearothermophilus* (*G. stearothermophilus*) spores and bacteriophage MS2 on N95 respirators, proposing a PAA room disinfection system as a solution for decontamination of a great number of N95 respirators during the outbreak of COVID-19 (14).

Peroxyacetic acid has been shown to be effective against food-borne pathogens on different produce surfaces (15, 16). Van de Velde et al. demonstrated the usefulness of PAA and H_2_O_2_ mixture for preserving the quality of strawberries by reducing the native microbial load on their surface (17). Another study assessed the effect of the disinfectant fogging procedure on the air quality in the farrowing-weaning room of a piggery. It was proven that fogging improved the quality of the air by reducing ammonia and dust concentration and the number of fungal spores. Therefore, the procedure resulted in the enhancement of the health of both the workers and animals (18). Our study adds new information to this topic, as we proved the effectiveness of fogging with PAA in schools and kindergartens–places where many children gather and spend the whole day performing various activities, such as eating, sleeping, playing.

Fogging disinfection may be safely used in kindergartens or environment of nurseries as the residues of the fog disappear in few hours, therefore, fogging may be performed during the absence of the children in kindergarten (nights, weekends), as the environment is completely safe the next day. Although most of the bacteria cultured from kindergartens were saprophytes, some of them might pose a threat to health of the children, especially to those immunocompromised. Thus, decontamination should regularly be performed to prevent the spreading of pathogens. The results presented in this study showed the high effectiveness of disinfection by fogging in both the schools and kindergartens.

In addition to bacteria, fungi such as *Aspergillus niger* (*A. niger*) and *Aspergillus flavus (A. flavus)* were cultured. According to the European standard PN-EN 13624, PAA can be classified as a fungicidal agent as it destroys the difficult-to-destroy spores of the fungus *A. niger* (now *Aspergillus brasiliensis*). In our study, *A. niger* spores were detected on the tables and walls of kindergartens and tables in schools. The effectiveness of PAA fogging was 100% in all the cases.

As a final discussion, we can emphasize that dry fog fumigation using PAA is a portable and simple decontamination technology for public spaces inhabited for large amount of time by known carriers of potentially harmful bacteria and fungi. For the study, kindergartens and schools were chosen since the public considers these places unique as children are largely either exposed or not to the pathogens in such environments. In conclusion, our study proves that decontamination by using this method is effective not only in the healthcare centers but also in the educational institutions. In times of the COVID-19 pandemic, effective, nontoxic, and regular disinfection of large places is important to ensure the safety of children and adolescents.

## Data Availability Statement

The raw data supporting the conclusions of this article will be made available by the authors, without undue reservation.

## Ethics Statement

The studies involving human participants were reviewed and approved by Medical University of Bialystok, Poland Bioethical Committee. Written informed consent for participation was not required for this study in accordance with the national legislation and the institutional requirements.

## Author Contributions

EK, HG, PC, SP, MG, and AM-M substantially contribution to conception and design or the acquisition and analysis of the data, and gave approval to the final submitted version. EK, PC, MG, MW, and AM-M drafted or critically revised the manuscript. All authors contributed to the article and approved the submitted version.

## Funding

This study was founded by the project No. POWR.03.02.00-00-I050/16 from the European Union funds, PO WER 2014-2020, grant No. 05/MSD/2019.

## Conflict of Interest

The authors declare that the research was conducted in the absence of any commercial or financial relationships that could be construed as a potential conflict of interest.

## Publisher's Note

All claims expressed in this article are solely those of the authors and do not necessarily represent those of their affiliated organizations, or those of the publisher, the editors and the reviewers. Any product that may be evaluated in this article, or claim that may be made by its manufacturer, is not guaranteed or endorsed by the publisher.
