# Corrigendum: Fogging With Peracetic Acid in Schools and Kindergartens

**DOI:** 10.3389/fpubh.2022.867618

**Published:** 2022-03-08

**Authors:** Ewelina Kruszewska, Henryk Grześ, Piotr Czupryna, Sławomir Pancewicz, Monika Groth, Mulugeta Wondim, Anna Moniuszko-Malinowska

**Affiliations:** ^1^Department of Infectious Diseases and Neuroinfections, Medical University of Bialystok, Bialystok, Poland; ^2^Microbiology Laboratory, University Clinical Hospital in Bialystok, Bialystok, Poland

**Keywords:** peracetic acid, fogging, decontamination, fumigation, automated

In the original article, the reference for 10 was incorrectly written as Fiorillo L, Cervino G, Matarese M, D'Amico C, Surace G, Paduano V, et al. COVID-19 surface persistence: a recent data summary and its importance for medical and dental settings. *Int J Environ Res Public Health*. (2020) 17:3132. doi: 10.3390/ijerph17093132. It should be Sher M, Mulder R. Comparison of aerosolized hydrogen peroxide fogging with a conventional disinfection product for a dental surgery. *J Contemp Dent Pract*. (2020) 21:1307–11.

The reference for 11 was incorrectly written as Krishnan J, Fey G, Stansfield C, Landry L, Nguy H, Klassen S, et al. Evaluation of a dry fogging system for laboratory decontamination. *Appl Biosaf*. (2012) 17:132–41. doi: 10.1177/153567601201700305. It should be Fiorillo L, Cervino G, Matarese M, D'Amico C, Surace G, Paduano V, et al. COVID-19 surface persistence: a recent data summary and its importance for medical and dental settings. *Int J Environ Res Public Health*. (2020) 17:3132. doi: 10.3390/ijerph17093132.

The reference for 12 was incorrectly written as Cutts T, Kasloff S, Safronetz D, Krishnan J. Decontamination of common healthcare facility surfaces contaminated with SARS-CoV-2 using peracetic acid dry fogging. *J Hosp Infect*. (2021) 109:82–7. doi: 10.1016/j.jhin.2020.12.016. It should be Krishnan J, Fey G, Stansfield C, Landry L, Nguy H, Klassen S, et al. Evaluation of a dry fogging system for laboratory decontamination. *Appl Biosaf*. (2012) 17:132–41. doi: 10.1177/153567601201700305.

The reference for 13 was incorrectly written as John AR, Raju S, Cadnum JL, Lee K, McClellan P, Akkus O, et al. Scalable in-hospital decontamination of N95 filtering face-piece respirator with a peracetic acid room disinfection system. *Infect Control Hosp Epidemiol*. (2020) 12:1–10. doi: 10.1017/ice.2020.1257. It should be Cutts T, Kasloff S, Safronetz D, Krishnan J. Decontamination of common healthcare facility surfaces contaminated with SARS-CoV-2 using peracetic acid dry fogging. *J Hosp Infect*. (2021) 109:82–7. doi: 10.1016/j.jhin.2020.12.016.

The reference for 14 was incorrectly written as Hilgren J, Swanson KM, Diez-Gonzalez F, Cords B. Inactivation of *Bacillus anthracis* spores by liquid biocides in the presence of food residue. *Appl Environ Microbiol*. (2007) 73:6370–77. doi: 10.1128/AEM.00974-07. It should be John AR, Raju S, Cadnum JL, Lee K, McClellan P, Akkus O, et al. Scalable in-hospital decontamination of N95 filtering face-piece respirator with a peracetic acid room disinfection system. *Infect Control Hosp Epidemiol*. (2020) 12:1–10. doi: 10.1017/ice.2020.1257.

The reference for 15 was incorrectly written as Vandekinderen I, Devlieghere F, De Meulenaer B, Ragaert P, Van Camp J. Optimization and evaluation of a decontamination step with peroxyacetic acid for fresh-cut produce. *Food Microbiol*. (2009) 26:882–88. doi: 10.1016/j.fm.2009.06.004. It should be Hilgren J, Swanson KM, Diez-Gonzalez F, Cords B. Inactivation of *Bacillus anthracis* spores by liquid biocides in the presence of food residue. *Appl Environ Microbiol*. (2007) 73:6370–77. doi: 10.1128/AEM.00974-07.

The reference for 16 was incorrectly written as Van de Velde F, Vaccari MC, Piagentini AM, Pirovani ME. Optimization of strawberry disinfection by fogging of a mixture of peracetic acid and hydrogen peroxide based on microbial reduction, color and phytochemicals retention. *Food Sci Technol Int*. (2016) 22:485–95. doi: 10.1177/1082013215625696. It should be Vandekinderen I, Devlieghere F, De Meulenaer B, Ragaert P, Van Camp J. Optimization and evaluation of a decontamination step with peroxyacetic acid for fresh-cut produce. *Food Microbiol*. (2009) 26:882–88. doi: 10.1016/j.fm.2009.06.004.

The reference for 17 was incorrectly written as Costa A, Colosio C, Gusmara C, Sala V, Guarino M. Effects of disinfectant fogging procedure on dust, ammonia concentration, aerobic bacterial and fungal spores in a farrowing-weaning room. *Ann Agric Environ Med*. (2014) 21:494–9. doi: 10.5604/12321966.1120589. It should be Van de Velde F, Vaccari MC, Piagentini AM, Pirovani ME. Optimization of strawberry disinfection by fogging of a mixture of peracetic acid and hydrogen peroxide based on microbial reduction, color and phytochemicals retention. *Food Sci Technol Int*. (2016) 22:485–95. doi: 10.1177/1082013215625696.

The reference for 18 was incorrectly written as Sher M, Mulder R. Comparison of aerosolized hydrogen peroxide fogging with a conventional disinfection product for a dental surgery. *J Contemp Dent Pract*. (2020) 21:1307–11. doi: 10.5005/jp-journals-10024-2983. It should be Costa A, Colosio C, Gusmara C, Sala V, Guarino M. Effects of disinfectant fogging procedure on dust, ammonia concentration, aerobic bacterial and fungal spores in a farrowing-weaning room. *Ann Agric Environ Med*. (2014) 21:494–9. doi: 10.5604/12321966.1120589.

The authors apologize for these errors and state that they do not change the scientific conclusions of the article in any way. The original article has been updated.

## Publisher's Note

All claims expressed in this article are solely those of the authors and do not necessarily represent those of their affiliated organizations, or those of the publisher, the editors and the reviewers. Any product that may be evaluated in this article, or claim that may be made by its manufacturer, is not guaranteed or endorsed by the publisher.
